# Different visual evoked potentials in neuromyelitis optica spectrum disorder-related optic neuritis and idiopathic demyelinating optic neuritis: a prospective longitudinal analysis

**DOI:** 10.1186/s12886-022-02595-5

**Published:** 2022-09-21

**Authors:** Cong Zheng, Ling Wang, Xiaoyu Xu, Manli Zhou, Kaiqun Liu, Yuxin Zhang, Xiujuan Zhao, Lin Lu, Wei Qiu, Xinyu Zhang, Hui Yang

**Affiliations:** 1 State Key Laboratory of Ophthalmology, Zhongshan Ophthalmic Center, Sun Yat-Sen University, Guangzhou, China; 2 Department of Ophthalmology, The Seventh Affiliated Hospital of Sun Yat-Sen University, Shenzhen, China; 3 Department of Neurology, The Third Affiliated Hospital of Sun Yat-Sen University, Guangzhou, China

**Keywords:** Neuromyelitis optica spectrum disorder, Idiopathic demyelinating optic neuritis, Visual evoked potential

## Abstract

**Background:**

To investigate different visual evoked potential (VEP) patterns in neuromyelitis optica spectrum disorder-related optic neuritis (NMOSD-ON) and idiopathic demyelinating optic neuritis (IDON).

**Methods:**

This was a longitudinal, prospective, case-control study. Eighty-four Chinese patients with acute optic neuritis were enrolled, including 26 NMOSD-ON patients and 58 IDON patients. All the patients underwent best-corrected visual acuity (BCVA) and full-field pattern reversal VEP recordings at the onset, 1 month, 3 months, and 6 months.

**Results:**

Within 15′ checks, the NMOSD-ON patients had more severe VEP amplitude reduction at 6 months (2.39 ± 4.63 μV vs. 6.96 ± 8.88 μV, *P* = 0.034). However, the IDON patients showed more frequently normal VEP response at 3 months (24.0% vs. 4.5%, *P* = 0.017), and only prolonged P100 peak latency with normal amplitude (L) at 6 months (30.0% vs. 57.8%, *P* = 0.048). Within 60′ checks, no significant difference in VEP parameters between the two groups was found at each follow-up (*P* > 0.05).

**Conclusions:**

The NMOSD-ON patients showed more severe axonal damage and worse axonal recovery than the IDON patients. VEP elicited by smaller check size was more sensitive to visual pathway abnormality in NMOSD-ON.

## Introduction

Optic neuritis (ON) is the most common manifestation and the first solitary clinical feature in neuromyelitis optica (NMO). With the detection of specific antibodies against aquaporin 4 (AQP4-Ab), NMO can be different from other demyelinating central nervous system (CNS) diseases [1]. NMO spectrum disease (NMOSD) was recently introduced to describe a broadened clinical spectrum [2]. Accordingly, ON with AQP4-Ab seropositivity was officially defined as NMOSD-ON when other diagnoses were excluded [3].

Visual evoked potential (VEP) is widely used in ON, reflecting demyelination and axonal damage in the visual pathway [4]. However, little is known about VEP changes in NMOSD-ON patients with an acute attack. Moreover, most previous studies [5] were retrospective or cross-sectional designs with inconsistent ON history, making it hard to draw a definite conclusion about the VEP in NMOSD-ON. With the development of novel NMOSD-ON treatments, it is necessary to describe the natural VEP pattern in NMOSD-ON using different check sizes.

This prospective follow-up study aimed to characterize the VEP pattern of NMOSD-ON and clarify the difference in VEP parameters between NMOSD-ON and IDON by using small 15′ checks and large 60′ checks.

## Materials and methods

### Subjects and patients

This longitudinal, prospective, case-control study was approved by the Institutional Review Board and Ethics Committee of Zhongshan Ophthalmic Center, Sun Yat-sen University, China(No. 2014 meky049). Written informed consent was obtained from all participants.

A total of 94 ON patients were recruited from Zhongshan Ophthalmic Center, Sun Yat-sen University, between November 2013 and October 2016. All participants’ detailed medical records included general medical history, routine ophthalmology examinations, magnetic resonance imaging (MRI) examination, and immunological tests.

The eligibility criteria were as follows: (1) meeting the diagnostic criteria of ON [6]; (2) within 30 days of an acute attack; (3) no relapse ≥ 90 days prior to the acute attack; (4) age of 18 years or older; (5) complete medical records of at least six months follow-up visits; (6) no brain lesions or myelitis; (7) at least 1 episode of clinical ON; (8) administration of IVMP treatment in previous attack and no other treatment.

The exclusion criteria were as follows: (1) other related ocular diseases affecting VEP, such as amblyopia and traumatic optic neuropathy; (2) positive in serum myelin oligodendrocyte glycoprotein antibody (MOG-Ab) testing.

### Group division

Among 94 patients, 10 patients had to be excluded from the study: 4 due to MOG-Ab-seropositivity, 4 lack of follow-up VEP records, and 2 with an ON history of more than one month. The remaining eighty-four patients were subdivided into two groups: Twenty-six AQP4-Ab-positive patients were diagnosed with NMOSD according to the 2015 diagnostic criteria (3), and fifty-eight AQP4-Ab-negative patients were diagnosed with IDON.

### Clinical assessment

BCVA was measured by Snellen charts and transformed into the logarithm of the minimum angle of resolution (logMAR). Finger count (FC), hand motion (HM), light perception (LP), and no light perception (NLP) were converted to 1.85, 2.0, 2.7, and 3.0, respectively [7].

Full-field pattern-reversal VEP was performed with Electrophysiological Diagnostic Systems (RETI-Port/Scan 21, ROLAND CONSULT Stasche & Finger GmbH, Germany). The examination procedure followed the International Society for Clinical Electrophysiology of Vision (ISCEV) standard [8]. Full-field monocular stimulation (stimulus contrast: 97%; check sizes: large 60′ and small 15′; pattern reversal rates: 1.8—2.2 reversals/s) by pattern reversal black or white checkerboards was performed at a viewing distance of 100 centimeter. Only P100 peak latencies and N75-P100 peak-to-peak amplitudes were analyzed. Only assessments performed with the same full-field pattern-reversal VEP and the same testing protocol at each follow-up were considered.

The upper limit of the normal distribution was commonly defined as 2 SDs above the mean [9]. VEP patterns were divided into five types: normal wave, only decreased amplitude with normal P100 latency (A), only prolonged P100 latency with normal amplitude (L), decreased amplitude with prolonged P100 latency (AL), and no wave. P100 peak latency was divided into five categories: normal, mild delay, moderate delay, severe delay, and no response. The classification of standards were as follows: (1) values within two SDs of the mean were defined as normal latency; (2) values of ≤  10 ms above normal values were defined as mild delay latency; (3) values between 10 ms to 20 ms above normal values were defined as moderate delay latency; (4) values of ≥  20 ms above normal values were defined as severe delay latency; (5) no wave detected was defined as no response.

Overall, BCVA and VEP were analyzed at the onset, 1 month, 3 months, and 6 months.

Laboratory tests were performed at baseline, including routine blood biochemical analysis, infectious test, and autoimmune test. Serum AQP4-IgG and MOG-IgG was tested by a cell-based assay (Euroimmun, Lübeck, Germany). The autoimmune examination was performed, including anti-thyroglobulin antibody, anti-thyroid peroxidase antibody, anti-nucleosome antibody, anti-histone antibody, anti-Sjögren’s-syndrome-related antigen A, anti-Sjögren’s-syndrome-related antigen B, anticardiolipin antibody, anti-neutrophil cytoplasmic antibody, and rheumatoid factor.

The orbital and craniocerebral MRI ( 3.0 T, DISCOVERY MR 750, GE Healthcare, United States) were performed to evaluate the lesions on optic nerves and exclude other diseases, such as tumors. MRI was performed at enrollment, which was restricted to T1-weighted imaging (T1WI), T2-weighted imaging, post-contrast T1WI sequences, and fat-suppressed sequences.

### Treatment

All patients received treatment with high-dose intravenous methylprednisolone (a daily dose of 1 g for 3 consecutive days) followed by oral prednisone tablets (starting at 1 - 2 mg/kg body weight per day) tapering off after at least 6 months. Prednisone was slowly tapered with 4 - 12 mg reductions every 7 - 10 days. In addition, prednisone (5 mg per day) and azathioprine (50 mg per day) was prescribed for maintenance treatment.

### Statistics analysis

All statistical analyses were performed with the SPSS statistical version 19.0 (SPSS Inc., Chicago, IL, USA). One eye was randomly included in the analysis for patients with bilateral ON. Continuous variables were presented as mean ± SD. Longitudinal analysis of VEP amplitudes was analyzed by independent-sample *t*-test. Comparison of VEP amplitude and logMAR BCVA between the NMOSD-ON group and the IDON group were analyzed by the Mann–Whitney *U* test. The Chi-square test or Fisher’s exact test was performed to compare differences among the categorical variables, such as gender, the affected eye, prior ON attacks, and wave pattern. Multiple linear regression was performed to analyze the relationship between logMAR BCVA and P100 amplitude. A *P*-value of < 0.05 was considered statistically significant.

## Results

### Patient characteristics (Table 1)

**Table 1:** Baseline characteristics of NMOSD-ON and IDON patients

	NMOSD-ON group (*n* = 26)	IDON group (*n* = 58)	*P*-value
Female, % (n)	84.6%(22)	55.2%(32)	0.009*
Age at onset (years), mean ± SD	31.54 ± 14.30	32.43 ± 17.02	0.805
Affected eye, % (n)			
Unilateral involvement	65.4%(17)	62.1%(36)	0.812
Bilateral involvement	34.6%(9)	37.9%(22)	
Previous attacks of ON, % (n)			
None	46.2%(12)	77.6%(45)	0.003*
Once	19.2%(5)	15.5%(9)	
More than once	34.6%(9)	6.9%(4)	
Disease duration (years), mean ± SD	3 ± 0.68	2.8 ± 0.60	0.310
Previous multifocal involvement			
Brain, %(n)	1	0	-
Spinal cord, %(n)	0	0	-
Time from onset to IVMP( days), mean ± SD	9.06 ± 7.30	8.80 ± 7.76	0.852
LogMAR BCVA at onset, mean ± SD	2.21 ± 1.54	1.87 ± 1.58	0.266
VEP amplitude of P100 at onset (μV), mean ± SD			
60′ check	2.14 ± 3.52	2.79 ± 5.33	0.847
15′ check	1.76 ± 2.65	3.35 ± 6.81	0.912
VEP latency			
VEP latency with 60′ check at onset, % (n)			
Normal	10.5% (2)	16.7% (7)	0.280
Mild delay	0 (0)	7.1% (3)	
Moderate delay	15.8% (3)	2.4% (1)	
Severe delay	5.3% (1)	9.5% (4)	
No response	68.4% (13)	64.3% (27)	
VEP latency with 15′ check at onset, % (n)			
Normal	5.3% (1)	11.9% (5)	0.663
Mild delay	0 (0)	2.4% (1)	
Moderate delay	5.3% (1)	7.1% (3)	
Severe delay	26.3% (5)	11.9% (5)	
No response	63.2% (12)	66.7% (28)	

*ON* Optic neuritis, *BCVA* Best corrected visual acuity, *VEP* Visual evoked potential, *NMOSD-ON* Neuromyelitis optica spectrum disorder-related optic neuritis, *IDON* Idiopathic demyelinating optic neuritis, *IVMP* Intravenous methylprednisolone

* Statistically significant (*P* < 0.05)

Twenty-six of the 84 patients were diagnosed with NMOSD-ON, and the remaining 58 patients were diagnosed with IDON. Female preponderance in the NMOSD-ON group was significantly higher than in the IDON group (84.6% vs. 55.2%, *P* = 0.009). The frequency of previous episodes was significantly higher in the NMOSD-ON group than in the IDON group (34.6% vs. 6.9%, *P* = 0.003). There were no significant differences in other baseline characteristics between the NMOSD-ON and IDON groups (*P* > 0.05).

### Longitudinal changes of logMAR BCVA (Table 2)

**Table 2:** Comparison of log MAR BCVA, VEP amplitude and VEP latency change between the NMOSD-ON and IDON patients

	NMOSD-ON group	IDON group	*P*-value
Log MAR BCVA, mean ± SD			
At Month 1	-1.20 ± 1.39	-0.71 ± 1.35	0.142
At Month 3	-1.25 ± 1.26	-1.05 ± 1.51	0.377
At Month 6	-1.17 ± 1.32	-1.04 ± 1.57	0.582
P (Month 1 vs. Onset)	< 0.001*	< 0.001*	-
P (Month 3 vs. Month 1)	0.921	0.001*	-
P (Month 6 vs. Month 3)	0.865	0.938	-
VEP amplitude changes with 60′ check, mean ± SD, μV			
At Month 1	2.27 ± 3.62	4.66 ± 6.78	0.901
At Month 3	3.24 ± 5.41	7.62 ± 8.15	0.060
At Month 6	3.42 ± 4.51	7.12 ± 6.61	0.059
*P* (Month 1 vs. Onset)	0.029*	0.001*	-
*P* (Month 3 vs. Month 1)	0.557	0.025*	-
*P* (Month 6 vs. Month 3)	0.461	0.255	-
VEP amplitude changes with 15′ check, mean ± SD, μV			
At Month 1	2.75 ± 6.31	3.73 ± 7.37	0.945
At Month 3	3.27 ± 6.16	5.72 ± 9.11	0.470
At Month 6	2.39 ± 4.63	6.96 ± 8.88	0.034*
*P* (Month 1 vs. Onset)	0.438	0.008*	-
*P* (Month 3 vs. Month 1)	0.278	0.002*	-
*P* (Month 6 vs. Month 3)	0.230	0.127	-
VEP latency with 60′ check, % (n)			
At Month 1			
Normal	11.1% (2)	13.6% (6)	0.776
Mild delay	27.8% (5)	15.9% (7)	
Moderate delay	22.2% (4)	20.5% (9)	
Severe delay	5.6% (1)	15.9% (7)	
No response	33.3% (6)	34.1% (15)	
At Month 3			
Normal	9.1% (2)	32.0% (16)	0.138
Mild delay	13.6% (3)	20.0% (10)	
Moderate delay	18.2% (4)	8.0% (4)	
Severe delay	22.7% (5)	20.0% (10)	
No response	36.4% (8)	20.0% (10)	
At Month 6			
Normal	25.0% (5)	33.3% (15)	0.067
Mild delay	5.0% (1)	31.1% (14)	
Moderate delay	15.0% (3)	6.7% (3)	
Severe delay	30.0% (6)	15.6% (7)	
No response	25.0% (5)	13.3% (6)	
*P* (Month 1 vs. Onset)	0.103	0.015*	-
*P* (Month 3 vs. Month 1)	0.544	0.085	-
*P* (Month 6 vs. Month 3)	0.537	0.715	-
VEP latency with 15′ check, % (n)			
At Month 1			
Normal	0 (0)	13.6% (6)	0.074
Mild delay	33.3% (6)	6.8% (3)	
Moderate delay	16.7% (3)	15.9% (7)	
Severe delay	16.7% (3)	25.0% (11)	
No response	33.3% (6)	38.6% (17)	
At Month 3			
Normal	9.1% (2)	24.0% (12)	0.435
Mild delay	9.1% (2)	12.0% (6)	
Moderate delay	13.6% (3)	12.0% (6)	
Severe delay	27.3% (6)	30.0% (15)	
No response	40.9% (9)	22.0% (11)	
At Month 6			
Normal	15.0% (3)	15.6% (7)	0.102
Mild delay	10.0% (2)	17.8% (8)	
Moderate delay	5.0% (1)	28.9% (13)	
Severe delay	30.0% (6)	20.0% (9)	
No response	40.0% (8)	17.8% (8)	
*P* (Month 1 vs. Onset)	0.033*	0.108	-
*P* (Month 3 vs. Month 1)	0.262	0.329	-
*P* (Month 6 vs. Month 3)	0.883	0.206	-

*BCVA* Best corrected visual acuity, *VEP* Visual evoked potential, *NMOSD-ON* Neuromyelitis optica spectrum disorder-related optic neuritis, *IDON* Idiopathic demyelinating optic neuritis, *IVMP* Intravenous methylprednisolone

* Statistically significant (*P* < 0.05)

BCVA in the NMOSD-ON group showed a significant improvement occurred at 1 month (*P* < 0.001), whereas that in the IDON group showed a similar improvement at 1 month and 3 months (*P* < 0.001, *P* = 0.001, respectively). However, there was no significant difference in BCVA between the two groups at any time point (*P*-values at 1, 3, and 6 months were 0.142, 0.377, and 0.582, respectively).

### Longitudinal changes of N75-P100 amplitudes (Table 2)

P100 amplitude of 60′ checks in the NMOSD-ON group increased significantly at 1 month (*P* = 0.029), whereas that in the IDON group increased significantly at 1 and 3 months ( *P*-values were 0.001 and = 0.025, respectively). However, there was no significant difference in P100 amplitude of 60′ checks between the two groups at any time point (*P*-values at 1, 3, and 6 months were 0.901, 0.060, and 0.059, respectively).

P100 amplitude of 15′ checks in the NMOSD-ON group showed no significant improvement at each follow-up, whereas that in the IDON group increased significantly at 1 month and 3 months (*P*-values were 0.008 and 0.002, respectively). However, P100 amplitude of 15′ checks in the NMOSD-ON group was significantly lower than in the IDON group at 6 months (*P* = 0.034).

### Longitudinal changes of P100 latency (Table 2)

No significant difference in P100 latency between the NMOSD-ON group and the IDON group was found at each time point (*P*-values of 60′ checks at 1, 3, and 6 months were 0.776, 0.138, 0.067, respectively; (*P*-values of 15′ checks at 1, 3, and 6 months were 0.074, 0.435, and 0.102, respectively).

### Longitudinal changes of VEP pattern (Table 3)

**Table 3:** Comparison of VEP pattern in NMOSD-ON and IDON patients

	NMOSD-ON group	IDON group	*P*-value
60′ check			
At onset			
Normal wave	10.5%(2)	14.3%(6)	1.000
A	0(0)	2.4%(1)	
L	15.8%(3)	14.3%(6)	
AL	5.3%(1)	4.8%(2)	
No wave	68.4%(13)	64.3%(27)	
At Month 1			
Normal wave	11.1%(2)	11.4%(5)	1.000
A	0(0)	4.5%(2)	
L	50.0%(9)	43.2%(19)	
AL	5.6%(1)	6.8%(3)	
No wave	33.3%(6)	34.1%(15)	
At Month 3			
Normal wave	9.1%(2)	30.1%(15)	0.219
A	4.5%(1)	2.0%(1)	
L	45.5%(10)	40.0%(20)	
AL	4.5%(1)	8.0%(4)	
No wave	36.4%(8)	16.95%(10)	
At Month 6			
Normal wave	25.0%(5)	31.1%(14)	0.771
A	0(0)	4.4%(2)	
L	40.0%(8)	42.2%(19)	
AL	10.0%(2)	8.9%(4)	
No wave	25.0%(5)	13.3%(6)	
*P* (Month 1 vs. Onset)	-	0.029*	-
*P* (Month 3 vs. Month 1)	0.918	0.189	-
*P* (Month 6 vs. Month 3)	0.486	0.889	-
15′ check			
At onset			
Normal wave	0%(0)	7.1%(3)	0.468
A	5.3%(1)	2.4%(1)	
L	21.1%(4)	21.4%(9)	
AL	10.5%(2)	2.4%(1)	
No wave	63.2%(12)	66.7%(28)	
At Month 1			
Normal wave	0(0)	11.4%(5)	0.565
A	0(0)	2.3%(1)	
L	44.4%(8)	34.1%(15)	
AL	22.2%(4)	13.6%(6)	
No wave	33.3%(6)	38.6%(17)	
At Month 3			
Normal wave	4.5%(1)	24.0%(12)	0.017*
A	4.5%(1)	0(0)	
L	36.4%(8)	52.0%(26)	
AL	13.6%(3)	2.0%(1)	
No wave	40.9%(9)	22.0%(11)	
At Month 6			
Normal wave	15.0%(3)	15.6%(7)	0.129
A	0(0)	0(0)	
L	30.0%(6)	57.8%(27)	
AL	15.0%(3)	8.9%(4)	
No wave	40.0% (8)	17.8% (8)	
*P* (Month 1 vs. Onset)	-	0.058	-
*P* (Month 3 vs. Month 1)	0.669	0.008*	-
*P* (Month 6 vs. Month 3)	0.689	-	-

*VEP* Visual evoked potential, *A* decreased N75-P100 peak-to-peak amplitude with normal P100 peak latency, *L* only prolonged P100 peak latency with normal amplitude, *AL* decreased N75-P100 peak-to-peak amplitude with prolonged P100 peak latency, *NMOSD-ON* Neuromyelitis optica spectrum disorder-related optic neuritis, *IDON* Idiopathic demyelinating optic neuritis, *IVMP* Intravenous methylprednisolone

* Statistically significant (*P* < 0.05)

Normal VEP responses of 15′ checks in the IDON group were significantly more frequent than in the NMOSD-ON group at 3 months (24.0% vs. 4.5%, *P* = 0.017), while no significant group difference in a normal VEP response of 60′ checks was found at each follow-up (*P*-values at 1, 3, and 6 months were 1.000, 0.219, and 0.770, respectively).

VEP patterns with A responses in the NMOSD-ON group (0—5.3%) and the IDON group (0—4.5%) were rare. L pattern response of 15′ checks in the IDON group was significantly more frequent than in the NMOSD-ON group at 6 months (30.0% vs. 57.8%, *P* = 0.048), while no statistical group difference in L pattern response of 60′ checks was found at any time point (*P*-values at 1, 3, and 6 months were 0.780, 0.807, and 0.255, respectively). Regarding those VEP patterns with AL, no statistical difference was found at any follow-up with neither 60′ checks (*P*-values at 1, 3, and 6 months were 1.000, 1.000, and 1.000, respectively) nor 15′ checks (*P*-values at 1, 3, and 6 months were 0.457, 0.082, and 0.667, respectively).

In terms of absent VEP response, no statistical difference between the two groups was observed at any follow-up (*P*-values of 60′ checks at 1, 3, and 6 months were 1.000, 0.076, and 0.292, respectively;* P*-values of 15′ checks at 1, 3, and 6 months were 0.145, 0.152, and 0.068, respectively).

### Relationship between logMAR BCVA and P100 amplitude (Table 4)

**Table 4:** Multivariable linear regression analyses for best-corrected visual acuity and P100 amplitude

Variable	LogMAR BCVA in the NMOSD-ON group		LogMAR BCVA in the IDON group	
	SPRC	*P*	SPRC	*P*
P100 amplitudes (60′ check)	-0.416	< 0.001*	-0.104	< 0.001*
P100 amplitudes (15′ check)	-0.317	< 0.001*	-0.014	0.208

*NMOSD-ON* Neuromyelitis optica spectrum disorder-related optic neuritis, *IDON* Idiopathic demyelinating optic neuritis, *IVMP* Intravenous methylprednisolone, *BCVA* Best-corrected visual acuity, *SPRC* Standardized partial regression coefficient

* Statistically significant (*P* < 0.05)

LogMAR BCVA in the NMOSD-ON group negatively correlated with P100 amplitudes of both the two checks (*P* < 0.001), whereas that in the IDON group only showed a negative correlation with P100 amplitudes of 60′ checks (*P* < 0.001) and did not correlate with P100 amplitudes of 15′ checks (*P* = 0.208). In comparison with the standardized partial regression coefficient (SPRC) of 60′ checks (SPRC = -0.416) and 15′ checks (SPRC = -0.317), the P100 amplitude of 60′ checks had a relatively greater influence on logMAR BCVA than that of 15′ checks.

## Discussion/conclusion

This prospective longitudinal study provided the detailed VEP changes between NMOSD-ON and IDON in the whole acute phase, recorded by 60′ checks and 15′ checks. Our results demonstrated that the NMOSD-ON patients have more severe axonal damage than the IDON patients regarding the P100 amplitude and abnormal VEP response pattern.

### Amplitudes and latency

VEP amplitudes have been considered to reflect the number of functional optic nerve fibers [10]. A significant reduction of amplitudes in the NMOSD-ON has been reported previously for at least three months after the onset [11]. According to our results, P100 amplitude of 15′ checks in the NMOSD-ON group showed a significant reduction at 6 months than in the IDON group. This result suggested more severe axonal damage in NMOSD-ON than in IDON.

Latency prolongation has been reported to reflect the demyelination of the optic nerve [12]. Delayed latency might remain abnormal for years after standard visual functions recovered. Our study showed no significant group difference in latency categories during follow-up, suggesting no difference in delayed latency severity between NMOSD-ON and IDON.

### VEP pattern

Absent VEP response more frequently occurred in NMOSD-ON at the onset ( nearly 10.7% [5]–62.5% [13] in previous studies). Our study showed a lack of VEP response was at least 60%. However, no significant difference in absent VEP recordings between the two groups was found, in consistent with a study by Marius Ringelstein et al. [5] that suggested heterogeneous patterns in NMO.

The dominant VEP abnormality pattern in both the NMOSD-ON and the IDON groups was latency prolongation. More delayed latency occurred in the IDON group than those in the NMOSD-ON group [14]. In this study, the latency prolongation of 15′ checks in the NMOSD group was significantly less frequent than in the IDON group at 6 months. Few patients had only an amplitude decrease pattern, which contradicts the so-called “NMO VEP pattern” reported by Neto, S. P et al. [15]. However, this inconsistency results due to ethnic differences or study design was still unknown.

### Recovery

In this study, the recovery time window of the NMOSD-ON group was one month, whereas that of the IDON group was at least three months. Such a short recovery window indicated that more prompt treatment in NMOSD-ON is needed. The time window for IDON in this study was consistent with previous studies of mixed ON groups [10]. However, there were other suggestions for a recovery window of one year [16], or four months [17] in another retrospective ON study with small samples.

### Sensitivity

In this study, 15′ checks were more sensitive than 60′ checks in demonstrating the differences in VEP abnormalities. As previously reported, different volumes of the visual cortex were activated by 60′ checks and 15′ checks [18]. In healthy subjects, smaller stimulus check fields (15′ checks) may demonstrate a higher amplitude of VEP and a more central visual field response, which means more sensitivity to macular-disc bundle change [18].

There were several limitations in this study. Firstly, the small sample size was insufficient to confirm statistical differences between NMOSD-ON and IDON. Secondly, VEP recovery beyond six months after onset and more VEP characteristics, including VEP changes between the affected eyes and the unaffected eyes, correlation factors in VEP latency, and the relationship between amplitude and RNFL thickness was unknown.

In conclusion, our study demonstrated that more abnormal VEP was in the NMOSD-ON than in the IDON, which suggested more severe axonal damage along the optic nerve. Follow-up analysis suggested that NMOSD-ON patients have worse recovery. Furtherly, a small check size was more sensitive to detecting abnormality in NMOSD-ON than a large check size.

## Data Availability

The data used to support the findings of this study are available from the corresponding author upon request.

## Abbreviations

VEP: Visual evoked potential
NMOSD-ON: Neuromyelitis optica spectrum disorder-related optic neuritis
IDON: Idiopathic demyelinating optic neuritis
BCVA: Best-corrected visual acuity
ON: Optic neuritis
NMO: Neuromyelitis optica
AQP4-IgG: Anti-aquaporin 4 antibody
CNS: Demyelinating central nervous system
NMOSD: NMO spectrum disease
MRI: Magnetic resonance imaging
logMAR: Logarithm of the minimum angle of resolution
FC: Finger count
HM: Hand motion
LP: Light perception
NLP: No light perception
ISCEV: International Society for Clinical Electrophysiology of Vision
SD: Standard deviations
SPRC: Standardized partial regression coefficient
T1WI: T1-weighted imaging
